# Multiregional MRI-based deep learning radiomics to predict axillary response after neoadjuvant chemotherapy in breast cancer patients

**DOI:** 10.1186/s13244-025-02193-1

**Published:** 2026-01-26

**Authors:** Weiyue Chen, Guihan Lin, Yi Zhou, Yongjun Chen, Changsheng Shi, Ting Zhao, Zhihan Yan, Zhiyi Peng, Shuiwei Xia, Min Xu, Minjiang Chen, Chenying Lu, Jiansong Ji

**Affiliations:** 1 Zhejiang Key Laboratory of Imaging and Interventional Medicine, The Fifth Affiliated Hospital of Wenzhou Medical University, Lishui, China; 2 Department of Radiology, Lishui Central Hospital, The Fifth Affiliated Hospital of Wenzhou Medical University, Lishui, China; 3 Department of Breast Surgery, The Fifth Affiliated Hospital of Wenzhou Medical University, Lishui, China; 4 Department of Radiology, The Sixth Affiliated Hospital of Wenzhou Medical University, Lishui, China; 5 Department of Interventional Vascular Surgery, The Third Affiliated Hospital of Wenzhou Medical University, Wenzhou, China; 6 Wenzhou Key Laboratory of Structural and Functional Imaging, Wenzhou, China; 7 Department of Radiology, The First Affiliated Hospital, Zhejiang University School of Medicine, Hangzhou, China

**Keywords:** Breast cancer, Deep learning radiomics, Axillary lymph node, Neoadjuvant chemotherapy, Peritumoral

## Abstract

**Objectives:**

This study was designed to develop a multiregional MRI-based deep learning radiomics nomogram (DLRN) for predicting axillary pathological complete response (apCR) after neoadjuvant chemotherapy (NAC) in breast cancer.

**Materials and methods:**

In total, 539 patients in our hospital were randomly split into a training cohort (TC; *n* = 431) and an internal validation cohort (IVC; *n* = 108), and 703 patients were recruited from three external centers as external validation cohorts (EVC1–3). Uni- and multivariate analyses were performed to select clinicopathological characteristics and establish a clinical model. DLR models were constructed based on DL and handcrafted radiomics features extracted from gross tumor volume (GTV) and GTV incorporating 3-, 5-, 7-, and 9-mm peritumoral regions (GPTV_3_, GPTV_5_, GPTV_7_, and GPTV_9_, respectively). A DLRN model incorporating the optimal DLR model and clinicopathological predictors was developed. Model performance was assessed employing the area under the receiver operating characteristic curve (AUC), calibration curve, and decision curve analysis.

**Results:**

The GPTV_5__DLR model surpassed the other DLR models, with an average AUC of 0.876 in the validation cohorts. The DLRN model better predicted apCR after NAC than the clinical model, demonstrating superior AUCs of 0.958 in the TC, 0.906 in the IVC, and 0.876–0.911 in EVC1–3. It also showed improved accuracy and clinical benefits for apCR prediction. Furthermore, the DLRN model achieved robust performance across different age, menstrual status, and clinical stage subgroups.

**Conclusion:**

The DLRN model, based on the GPTV_5__DLR model and clinicopathological features, exhibited high predictive efficiency for apCR after NAC.

**Critical relevance statement:**

The deep learning radiomics nomogram based on intra- and peritumoral regions could noninvasively predict axillary pCR in breast cancer patients receiving NAC, which might prevent patients from undergoing unnecessary axillary lymph node dissection.

**Key Points:**

- Combining intratumoral and 5-mm peritumoral region radiomics had the highest predictive efficiency for axillary pCR after NAC in breast cancer.
- The deep learning radiomics nomogram based on intra- and peritumoral regions outperformed the clinical model.
- The proposed model could provide a noninvasive and easy-to-use tool to offer decision support for optimizing treatments.

**Graphical Abstract:**

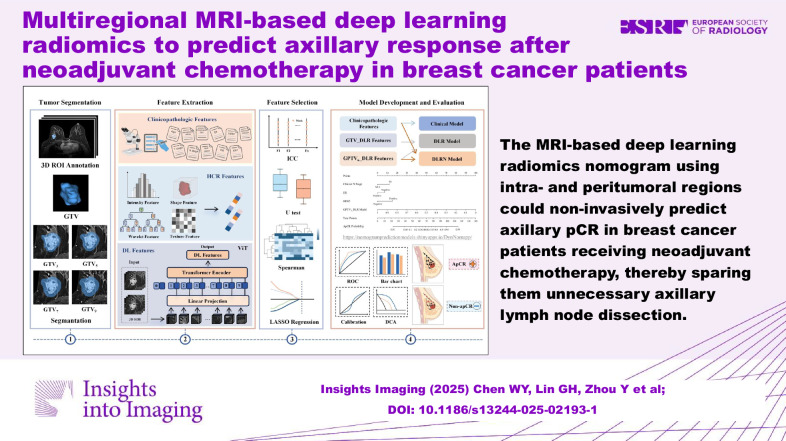

## Introduction

Breast cancer is the most common malignant tumor and the main cause of cancer‐related deaths among women [1]. Neoadjuvant chemotherapy (NAC) constitutes the principal therapeutic modality for locally advanced and triple-negative breast cancer, offering the advantages of reduced tumor burden, increased feasibility of breast‐conserving surgery and axillary procedures, and enhanced survival outcomes in patients achieving pathological complete response (pCR) in their breast or axilla [2]. Research has shown that 50–60% of patients with axillary lymph node (ALN) metastases achieve axillary pCR (apCR) following NAC [3]. In patients who achieve apCR, the omission of axillary lymph node dissection (ALND) can guard against related morbidities like a restricted range of motion and arm pain [4, 5]. Sentinel lymph node biopsy is a less invasive alternative to ALND; however, its false-negative rate (FNR) exceeds 10%, especially when fewer than three sentinel lymph nodes are excised [4, 6]. Furthermore, for breast cancer patients with ALN metastasis, targeted axillary dissection following NAC is increasingly being considered. However, challenges remain in terms of the detection rate and FNR [7–9]. Consequently, a reliable, noninvasive model is urgently needed to assess ALN response to NAC and guide the selection of less invasive surgical strategies.

Several imaging modalities (like ultrasonography, CT, and mammography) are utilized for monitoring ALN response to NAC [10–12]. In addition, contrast-enhanced mammography has emerged as a promising technique, offering specific advantages in detecting residual breast lesions [13, 14]. Although these techniques are noninvasive, the detection of micrometastases within small lymph nodes remains challenging. MRI, with its superior spatial resolution, allows for the all-sided delineation of whole breast carcinoma lesions and affords detailed assessments of ALN status. However, significant interobserver variability among radiologists underscores its inconsistent sensitivity and specificity for identifying ALN metastases [15–17].

Recently, radiomics has objectively quantified tumor heterogeneity via the high-throughput extraction of imaging features, thereby broadening the scope of conventional imaging applications [18]. Prior studies have demonstrated that MRI-based radiomics signatures represent promising noninvasive biomarkers for predicting apCR following NAC [19–21]. However, these investigations have predominantly focused on features extracted solely from gross tumor volume (GTV), ignoring possible alterations in peritumoral tissue that may compromise diagnostic accuracy. Emerging evidence indicates that the peritumoral region harbors valuable biological data related to tumor progression and metastasis, including lymphocytic infiltration, peritumoral lymphocytic invasion, and lymphangiogenesis [22–24]. More recently, radiomics features derived from the combined analysis of the GTV and gross peritumoral tumor volume (GPTV) have shown promise in diagnosis [25], lymph node metastasis prediction [26], and molecular subtype classification [27] in breast cancer, underscoring the importance of integrating peritumoral tissue analysis for comprehensive evaluation. Nevertheless, despite the availability of thousands of handcrafted radiomics (HCR) features, the full depth of textural information within images remains underexploited.

Deep learning (DL) transforms input information into higher-level representations via iterative learning and weight modulation. Compared with HCR features, DL features can capture more abstract information and may reveal additional predictive patterns. Huang et al [28] established a valid DL radiomics (DLR) framework that automatically extracts lesion-relevant deep semantic features from DL segmentation networks and integrates them via a feature selection module. This DLR framework, which synergizes radiomics with DL algorithms, mitigates overfitting and has exhibited excellent performance in several studies [29–32]. However, the use of DLR features for predicting apCR has not been reported thus far. Moreover, it remains unclear whether a DLR model based on GPTV can further enhance the prediction of apCR following NAC. The current work aimed to evaluate the potential of DLR features extracted from intra- and peritumoral regions for predicting apCR following NAC and compare their performance in accurately determining the optimal peritumoral region size.

## Materials and methods

### Study cohorts and participants

**Fig. 1:**
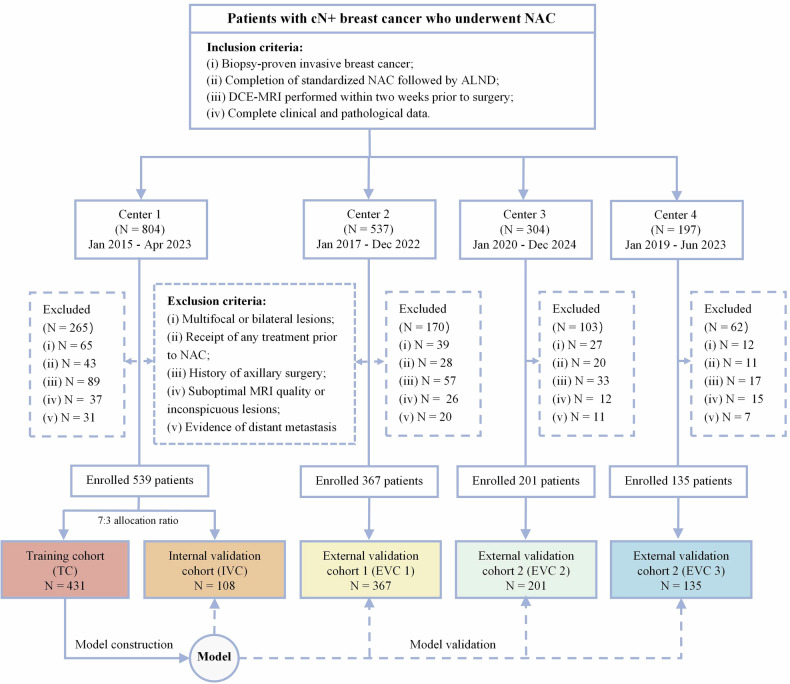
A flowchart illustrating the patient selection process across the four participating medical centers in this study

This research was approved by the Ethics Committee of Center 1 (Approval No. 2024-123). Given the study’s retrospective nature, the need for informed consent from participants was waived. Based on the inclusion and exclusion criteria (Appendix E1), a total of 1242 cN+ breast cancer patients who underwent NAC in four medical centers were recruited, as shown in Fig. 1. All patients were assigned to five cohorts: a training cohort (TC; *n* = 431 from Center 1 (the Fifth Affiliated Hospital at Wenzhou Medical University)), an internal validation cohort (IVC; *n* = 108 from Center 1), and three external validation cohorts (EVC1–3; *n* = 367 from Center 2 (the Sixth Affiliated Hospital of Wenzhou Medical University), *n* = 201 from Center 3 (the Third Affiliated Hospital of Wenzhou Medical University), and *n* = 135 from Center 4 (the First Affiliated Hospital of Zhejiang University)). NAC regimens and clinicopathological data collection are detailed in Appendix E2.

### MRI acquisition and tumor segmentation

MRI was performed using different MRI scanners at the four participating hospitals. Details of MRI image acquisition are provided in Appendix E3. Table S1 lists the specific parameters of the dynamic contrast-enhanced (DCE)-MRI sequences used by different MR scanners. The details of image preprocessing are shown in Appendix E4.

Two experienced radiologists independently delineated regions of interest (ROIs) layer by layer for GTV on third-phase images of the DCE-MRI sequence and then generated a 3D ROI. Segmentation was carried out semi-automatically utilizing ITK-SNAP software (version 3.8.0; itksnap.org). GTV was defined as a tumor confined within the visible boundaries on MRI. All segmentations were performed without access to pathological findings. Using the Simple ITK module in Python software (version 3.7.6), the GTV was outwardly expanded by 3, 5, 7, and 9 mm to generate GPTVs labeled GPTV_3_, GPTV_5_, GPTV_7_, and GPTV_9,_ respectively. These tumor regions were subsequently reviewed and manually adjusted to exclude non-tumor components like skin, chest wall, and air. The segmentation process is illustrated in Fig. S1.

### Image feature extraction

HCR features were extracted utilizing the open-source PyRadiomics (version 3.0.1; pyradiomics.readthedocs.io/en/v3.0.1) toolkit in Python. For each 3D ROI, 1218 HCR features were extracted, totaling 6090 features from five ROIs of different regions per patient. Detailed information on HCR feature extraction is given in Appendix E5. In this study, DL features were extracted utilizing the Vision Transformer proposed by Dosovitskiy et al [33]. Finally, 256 DL features were obtained for each 3D ROI, totaling 1280 per patient. Details of DL feature extraction are provided in Appendix E6.

### DLR feature selection

To eliminate differences in scale across feature dimensions, all HCR and DL features were standardized utilizing Z-score normalization and the ComBat method [34] (Appendix E7). The HCR feature reproducibility analysis and screening methods for all features are described concretely in Appendix E8. Ultimately, the selected HCR and DL features were combined, and the least absolute shrinkage and selection operator was employed to choose features included in the DLR model. This feature screening process was used to identify the most informative features associated with apCR after NAC in different regions.

### Model construction and visualization

**Fig. 2:**
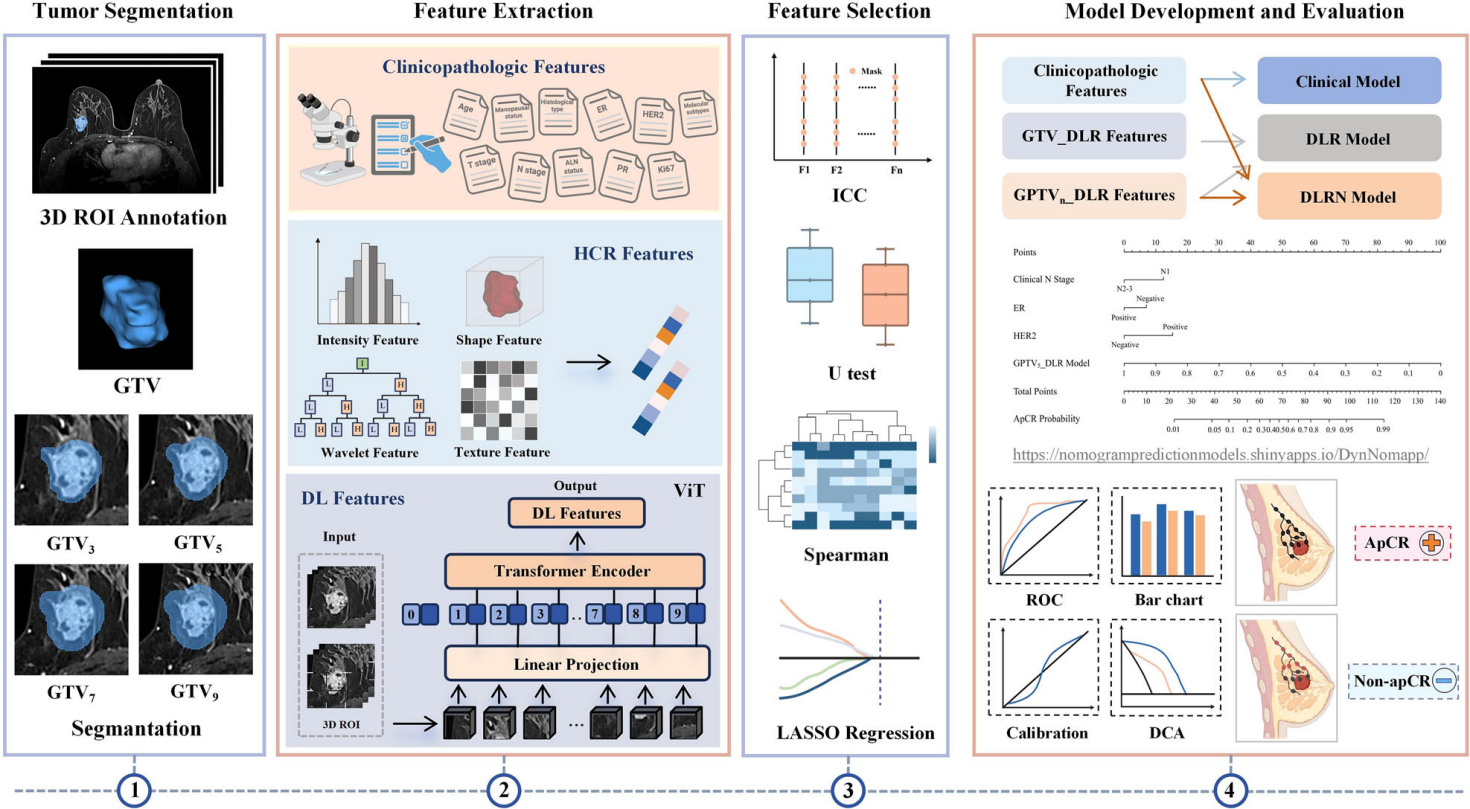
The study workflow. The four main phases included tumor segmentation, feature extraction, feature selection, and model development and evaluation

Multivariate logistic regression (LR) was used to develop region-specific DLR models. The DLR models’ predictive performance was assessed utilizing receiver operating characteristic (ROC) analysis. The best-performing DLR model with the highest average area under the curve (AUC) and accuracy in the IVC and EVC1–3 was selected for nomogram development. Feature visualization and analysis details for the optimal DLR model are provided in Appendix E9. Subsequently, univariate and multivariate LR analysis were performed to screen significant clinical predictors and establish a clinical model. A multivariate LR analysis was also utilized to construct a DLR nomogram (DLRN) by integrating the optimal DLR model and clinical predictors. The LR coefficients in the DLR and DLRN models were weighted to obtain the corresponding predicted scores. An overview of our study workflow is presented in Fig. 2.

### METhodological RadiomICs Score (METRICS)

The METRICS tool was developed by a large international group of experts to evaluate and improve the quality of radiomics research [35]. A web application has been developed to assist in calculating the METRICS score (https://metricsscore.github.io/metrics/METRICS.html). The METRICS score of our study was 97.7%, placing it in the excellent quality category. The completed checklists are provided in Appendix E10.

### Statistical analysis

Statistical analyses were performed using R (version 4.2) and Python (version 3.9.6). Student’s *t*-test or the Mann–Whitney U-test was employed to compare continuous variables between groups, while the chi-square test or Fisher’s exact test was used to compare categorical variables. The Delong test was utilized to compare various models’ AUCs. Calibration curves were generated using 1000 bootstrap resamples to assess alignment between predicted and actual risks. Decision curve analysis (DCA) could provide more reliable clinical benefit evidence. Subgroup analyses based on age, menopausal status, and clinical T and N stage were also conducted. All statistical tests were two-tailed; *p* < 0.05 denoted statistical significance.

## Results

### Patient features and clinical model

The patient clinicopathological characteristics of all cohorts are presented in Table 1. The mean age of the enrolled patients was 52.0 ± 10.7 years (between 21 and 85 years). The rates of apCR after NAC were 192 (44.5%), 48 (44.4%), 172 (46.9%), 97 (48.3%), and 58 (43.0%) in the TC, IVC, and EVC1–3 cohorts, respectively. The univariate and multivariate LR analysis revealed clinical N stage, estrogen receptor (ER), and human epidermal growth factor receptor (HER2) as independent predictors of apCR after NAC in the clinical model (Table S2).

**Table 1:** Clinicopathological characteristics of patients with breast cancer

Characteristics	TC (*n* = 431)	IVC (*n* = 108)	EVC1 (*n* = 367)	EVC2 (*n* = 201)	EVC3 (*n* = 135)
Age (years), *n* (%)					
≤ 45	163 (37.8)	40 (37.0)	127 (34.6)	65 (32.3)	37 (27.4)
> 45	268 (62.2)	68 (63.0)	240 (65.4)	136 (67.7)	98 (72.6)
Menopausal status, *n* (%)					
Premenopausal	240 (55.7)	57 (52.8)	215 (58.6)	114 (56.7)	74 (54.8)
Postmenopausal	191 (44.3)	51 (47.2)	152 (41.4)	87 (43.3)	61 (45.2)
Histological type, *n* (%)					
Invasive ductal carcinoma	391 (90.7)	96 (88.9)	320 (87.2)	188 (93.5)	124 (91.9)
Others	40 (9.3)	12 (11.1)	47 (12.8)	13 (6.5)	11 (8.1)
Clinical T stage, *n* (%)					
T1–2	275 (63.8)	76 (70.4)	221 (60.2)	137 (68.2)	94 (69.6)
T3–4	156 (36.2)	32 (29.6)	146 (39.8)	64 (31.8)	41 (30.4)
Clinical N stage, *n* (%)					
N1	316 (73.3)	74 (68.5)	285 (77.7)	145 (72.1)	103 (76.3)
N2–3	115 (26.7)	34 (31.5)	82 (22.3)	56 (27.9)	32 (23.7)
ER, *n* (%)					
Negative	185 (42.9)	47 (43.5)	177 (48.2)	85 (42.3)	66 (48.9)
Positive	246 (57.1)	61 (56.5)	190 (51.8)	116 (57.7)	69 (51.1)
PR, *n* (%)					
Negative	224 (52.0)	56 (51.9)	201 (54.8)	93 (46.3)	64 (47.4)
Positive	207 (48.0)	52 (48.1)	166 (45.2)	108 (53.7)	71 (52.6)
HER2, *n* (%)					
Negative	261 (60.6)	73 (67.6)	251 (68.4)	130 (64.7)	95 (70.4)
Positive	170 (39.4)	35 (32.4)	116 (31.6)	71 (35.3)	40 (29.6)
Ki-67, *n* (%)					
< 20	142 (32.9)	40 (37.0)	128 (34.9)	62 (30.8)	46 (34.1)
≥ 20	289 (67.1)	68 (63.0)	239 (65.1)	139 (69.2)	89 (65.9)
Molecular subtypes, *n* (%)					
HR+/HER2−	171 (39.7)	46 (42.6)	139 (37.9)	88 (43.8)	56 (41.5)
HER2+	191 (44.3)	48 (44.4)	166 (45.2)	166 (41.3)	57 (42.2)
TN	69 (16.0)	14 (13.0)	62 (16.9)	62 (14.9)	22 (16.3)
Post-NAC ALN status, *n* (%)					
apCR	192 (44.5)	48 (44.4)	172 (46.9)	97 (48.3)	58 (43.0)
Non-apCR	239 (55.5)	60 (55.6)	195 (53.1)	104 (51.7)	77 (57.0)

*ALN* axillary lymph node, *apCR* axillary lymph node pathological complete response, *ER* estrogen receptor, *EVC* external validation cohort, *HER2* human epidermal growth factor receptor 2, *HR* hormone receptor, *IVC* internal validation cohort, *NAC* neoadjuvant chemotherapy, *PR* progesterone receptor, *SD* standard deviation, *TC* training cohort, *TN* triple-negative

### DLR model building and evaluation

**Fig. 3:**
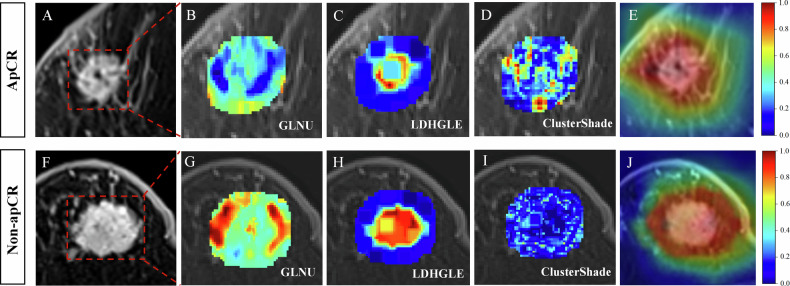
Visualization of the top three handcrafted radiomics (HCR) features and gradient-weighted class activation mapping (Grad-CAM) heat maps in (**A**) a 55-year-old female patient with axillary pathological complete response (apCR) after neoadjuvant chemotherapy (NAC), and (**F**) a 67-year-old female patient without apCR after NAC. **B**–**D**, **G**–**I** Pseudocolor overlays of the tumor with pixel intensity and the top three HCR features. The red and yellow regions indicate higher pixel feature values, whereas the blue and green regions represent lower pixel feature values. The texture patterns within the tumors of non-apCR patients compared to apCR patients show higher complexity, with notable variations in the distribution of gray levels, as indicated by gray-level non-uniformity (GLNU) (**B**, **G**). Regions with large gray-level dependence, indicating high contrast between different tumor regions, are emphasized by large dependence high gray-level emphasis (LDHGLE) (**C**, **H**). Moreover, tumors with more clustered gray-level distributions exhibit more pronounced texture variation, particularly in areas with higher contrast, as reflected by cluster shade (**D**, **I**). **E**, **J** Overlay of tumors and Grad-CAM heat maps of two patients. The corresponding heat maps demonstrate that the deep learning features that were weighted highest, representing the areas of greatest interest for the model, were located inside the intra- and peritumoral regions

DLR feature selection outcomes are given in Appendix E11 and Table S3. Based on the optimal λ values, we acquired 11, 13, 17, 13, and 9 DLR features, respectively, for further establishing DLR models (Fig. S2). The predicted-score formulas for the five DLR models based on multivariate LR are presented in Appendix E12. The comparison of their ROC curves, AUCs, and performance metrics is shown in Table 2 and Fig. S3. Among the DLR models for all regions, the GPTV_5_ model was selected as the optimal DLR model because it had a 0.876 average AUC and a 0.834 average accuracy in the IVC and EVC1–3. Additionally, the DeLong test showed that the GPTV_5_ model dramatically surpassed other DLR models in the TC (all *p* < 0.05) and performed well in the IVC and EVC1–3 (Table S4). Figure 3 visualizes the top three HCR features and gradient-weighted class activation mapping heat maps of two breast cancer patients.

**Table 2:** Performance of different DLR models for prediction of axillary lymph node pathological complete response to neoadjuvant chemotherapy in the TC, IVC, and EVC1–3

Cohorts	DLR models	AUC (95% CI)	Accuracy	Sensitivity	Specificity	PPV	NPV	Precision	Recall	F1-score
TC	GTV	0.872 (0.840–0.904)	0.789	0.781	0.795	0.754	0.819	0.754	0.781	0.767
	GPTV_3_	0.881 (0.847–0.916)	0.840	0.844	0.837	0.806	0.870	0.806	0.844	0.825
	GPTV_5_	0.925 (0.900–0.949)	0.875	0.859	0.887	0.859	0.887	0.859	0.859	0.859
	GPTV_7_	0.890 (0.858–0.922)	0.835	0.870	0.808	0.784	0.885	0.784	0.870	0.825
	GPTV_9_	0.833 (0.793–0.873)	0.803	0.776	0.824	0.780	0.821	0.780	0.776	0.778
IVC	GTV	0.827 (0.751–0.904)	0.778	0.792	0.767	0.731	0.821	0.731	0.792	0.760
	GPTV_3_	0.851 (0.779–0.924)	0.806	0.813	0.800	0.765	0.842	0.765	0.813	0.788
	GPTV_5_	0.887 (0.817–0.957)	0.861	0.833	0.883	0.851	0.869	0.851	0.833	0.842
	GPTV_7_	0.844 (0.770–0.918)	0.787	0.854	0.733	0.719	0.863	0.719	0.854	0.781
	GPTV_9_	0.861 (0.787–0.936)	0.815	0.833	0.800	0.769	0.857	0.769	0.833	0.800
EVC1	GTV	0.814 (0.770–0.857)	0.763	0.790	0.733	0.770	0.754	0.770	0.790	0.780
	GPTV_3_	0.845 (0.805–0.885)	0.801	0.805	0.797	0.818	0.783	0.818	0.805	0.811
	GPTV_5_	0.869 (0.831–0.907)	0.820	0.826	0.814	0.834	0.805	0.834	0.826	0.830
	GPTV_7_	0.778 (0.730–0.825)	0.755	0.779	0.727	0.764	0.744	0.764	0.779	0.771
	GPTV_9_	0.764 (0.715–0.812)	0.738	0.723	0.756	0.770	0.707	0.770	0.723	0.746
EVC2	GTV	0.837 (0.778–0.895)	0.816	0.825	0.808	0.800	0.832	0.800	0.825	0.812
	GPTV_3_	0.862 (0.809–0.916)	0.821	0.876	0.769	0.780	0.870	0.780	0.876	0.825
	GPTV_5_	0.893 (0.849–0.937)	0.841	0.887	0.798	0.804	0.883	0.804	0.887	0.843
	GPTV_7_	0.836 (0.780–0.891)	0.801	0.856	0.750	0.761	0.848	0.761	0.856	0.806
	GPTV_9_	0.793 (0.730–0.856)	0.786	0.794	0.779	0.770	0.802	0.770	0.794	0.782
EVC3	GTV	0.790 (0.710–0.871)	0.778	0.793	0.766	0.719	0.831	0.719	0.793	0.754
	GPTV_3_	0.815 (0.741–0.890)	0.800	0.759	0.831	0.772	0.821	0.772	0.759	0.765
	GPTV_5_	0.853 (0.784–0.921)	0.815	0.776	0.844	0.789	0.833	0.789	0.776	0.782
	GPTV_7_	0.807 (0.733–0.880)	0.763	0.793	0.740	0.697	0.826	0.697	0.793	0.742
	GPTV_9_	0.719 (0.629–0.809)	0.719	0.707	0.727	0.661	0.767	0.661	0.707	0.683

*AUC* area under the curve, *CI* confidence interval, *DLR* deep learning radiomics, *DLRN* deep learning radiomics nomogram, *EVC* external validation cohort, *GPTV* gross peritumoral tumor volume, *GTV* gross tumor volume, *IVC* internal validation cohort, *NPV* negative predictive value, *PPV* positive predictive value, *TC* training cohort

### DLRN model construction

**Fig. 4:**
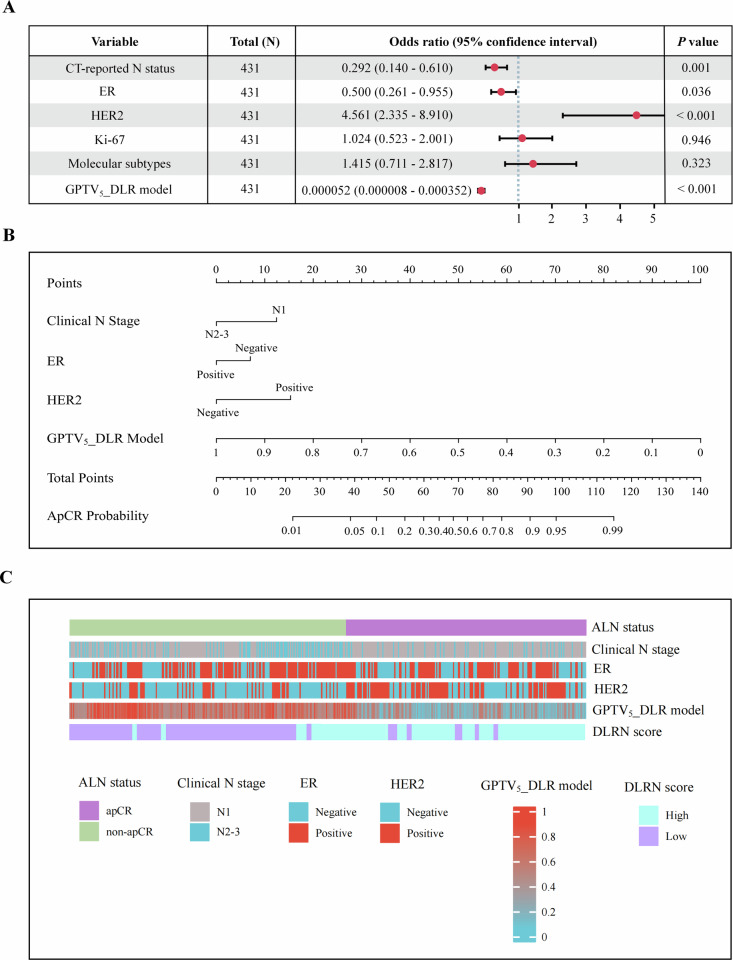
Construction of the deep learning radiomics nomogram (DLRN). **A** Forest plot of independent predictors in the training cohort. **B** The DLRN, scaled by the logistic regression coefficient of each independent predictor. **C** Heat map showing the association of axillary lymph node (ALN) status after NAC with the clinical N stage, estrogen receptor (ER), human epidermal growth factor receptor 2 (HER2), gross peritumoral tumor volume (GPTV)_5__deep learning radiomics (DLR) model, and DLRN score in the training cohort

The multivariate LR results showed that the clinical N stage, ER, HER2, and the GPTV_5__DLR model were independent predictors of apCR after NAC (Fig. 4A). We employed these predictors to establish the DLRN model and visualize a nomogram, as depicted in Fig. 4B. The DLRN score calculation formula, founded upon the nomogram, is given in Table S5. The DLRN score’s optimal cut-off value was 0.435 according to the Youden index, dividing all patients into low- and high-DLRN-score groups. Associations between individual predictors, DLRN scores, and ALN status are shown in Fig. 4C. An online calculator was created to determine the probability of apCR before NAC initiation by entering the relevant features (https://nomogrampredictionmodels.shinyapps.io/DynNomapp/).

### Performance comparison of different models

**Fig. 5:**
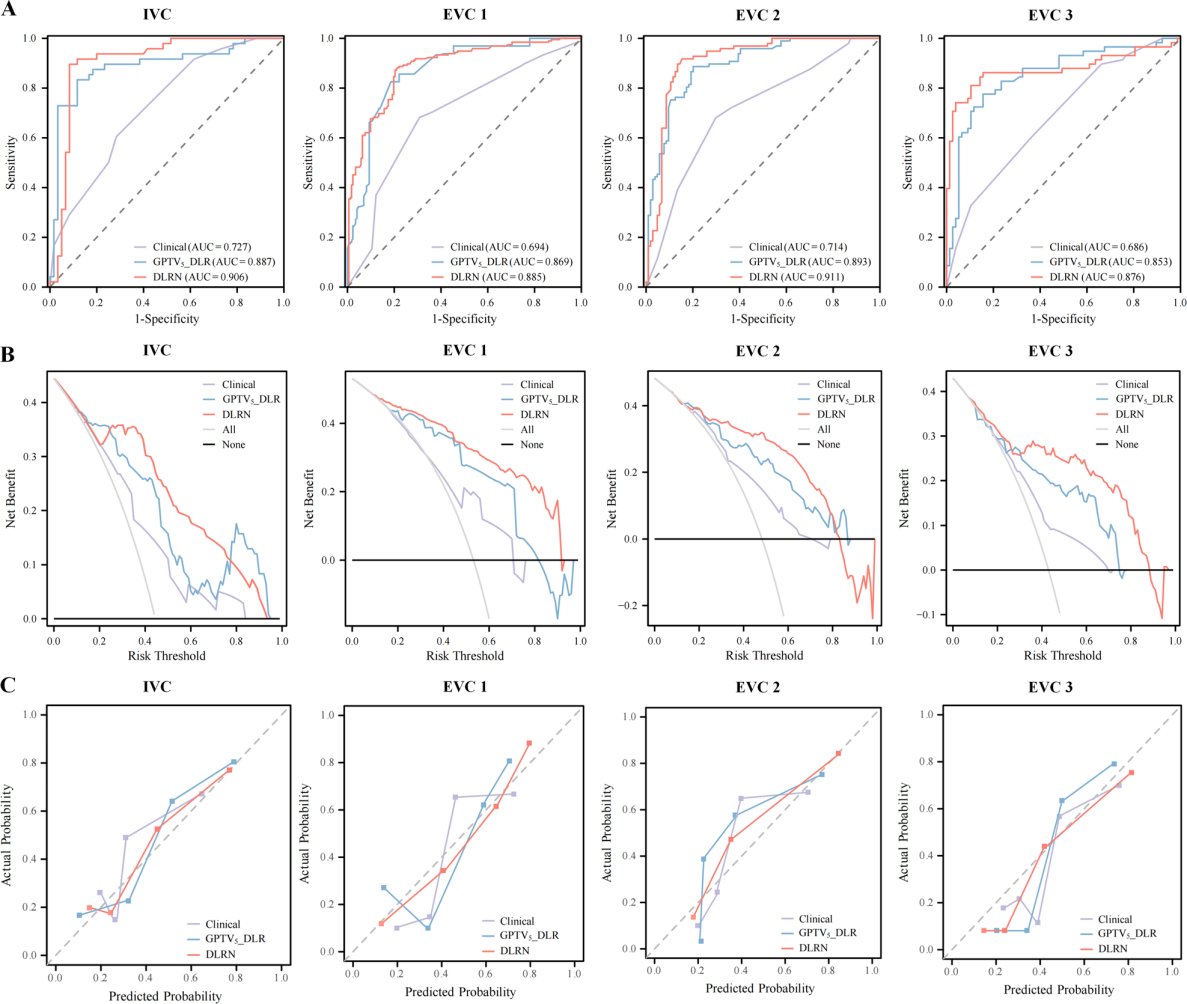
Comparison of the model’s performance in the internal validation cohort (IVC) and external validation cohorts 1–3 (EVC1–3). **A** Comparison of the receiver operating characteristic curves and areas under the curve (AUCs) of the clinical, gross peritumoral tumor volume (GPTV)_5__deep learning radiomics (DLR), and DLR nomogram (DLRN) models. **B** Comparison of the calibration curves of the clinical, GPTV_5__DLR, and DLRN models. **C** Comparison of the decision curves of the clinical, GPTV_5__DLR, and DLRN models

Significant differences in the GPTV_5__DLR and DLRN scores between apCR and non-apCR groups are shown in Fig. S4 (all *p* < 0.001). The performance metrics and ROC curves of the clinical, GPTV_5__DLR, and DLRN models for various cohorts are shown in Table 3 and Figs. 5A and S5A. Among the three models, the DLRN model realized the highest AUC of 0.958 (95% confidence interval (CI): 0.939–0.978) in the TC compared to the clinical and GPTV_5__DLR models (DeLong test, *p* < 0.001 and *p* = 0.042, respectively, Table S6). The DLRN model also exhibited outstanding performance in the IVC (AUC 0.906 [95% CI: 0.843–0.970]), EVC1 (AUC 0.885 [95% CI: 0.851–0.919]), EVC2 (AUC 0.911 [95% CI: 0.867–0.954]), and EVC3 (AUC 0.876 [95% CI: 0.806–0.946]), with no significant difference in AUCs between VCs (*p* = 0.363–0.911), indicating the model’s robustness. Furthermore, according to the DeLong test, the DLRN model exhibited better performance in predicting apCR before NAC initiation in breast cancer compared with the clinical model in all VCs (*p* < 0.05, Table S6). DCA plots indicated that the DLRN model generated more net benefit compared with the clinical and DLR models in all cohorts (Figs. 5B, S5B). The calibration curve showed that apCR probabilities predicted by the DLRN model had the best consistency with actual outcomes (Figs. 5C, S5C).

**Table 3:** Performance of different models for prediction of axillary lymph node pathological complete response to neoadjuvant chemotherapy in the TC, IVC, and EVC1–3

Cohorts	Models	AUC (95% CI)	Accuracy	Sensitivity	Specificity	PPV	NPV	Precision	Recall	F1-score	*p*-value*
TC	Clinical	0.746 (0.701–0.792)	0.701	0.510	0.854	0.737	0.685	0.737	0.510	0.603	< 0.001
	GPTV_5__DLR	0.925 (0.900–0.949)	0.875	0.859	0.887	0.859	0.887	0.859	0.859	0.859	0.042
	DLRN	0.958 (0.939–0.978)	0.930	0.922	0.937	0.922	0.937	0.922	0.922	0.922	-
IVC	Clinical	0.727 (0.636–0.819)	0.667	0.604	0.717	0.630	0.694	0.630	0.604	0.617	0.001
	GPTV_5__DLR	0.887 (0.817–0.957)	0.861	0.833	0.883	0.851	0.869	0.851	0.833	0.842	0.698
	DLRN	0.906 (0.843–0.970)	0.907	0.896	0.917	0.896	0.917	0.896	0.896	0.896	-
EVC1	Clinical	0.694 (0.640–0.747)	0.687	0.682	0.692	0.715	0.657	0.715	0.682	0.698	< 0.001
	GPTV_5__DLR	0.869 (0.831–0.907)	0.820	0.826	0.814	0.834	0.805	0.834	0.826	0.830	0.541
	DLRN	0.885 (0.851–0.919)	0.837	0.872	0.797	0.829	0.846	0.829	0.872	0.850	-
EVC2	Clinical	0.714 (0.644–0.785)	0.692	0.680	0.702	0.680	0.702	0.680	0.680	0.680	< 0.001
	GPTV_5__DLR	0.893 (0.849–0.937)	0.841	0.887	0.798	0.804	0.883	0.804	0.887	0.843	0.579
	DLRN	0.911 (0.867–0.954)	0.881	0.918	0.846	0.848	0.917	0.848	0.918	0.882	-
EVC3	Clinical	0.686 (0.599–0.773)	0.622	0.603	0.636	0.556	0.681	0.556	0.603	0.579	0.001
	GPTV_5__DLR	0.853 (0.784–0.921)	0.815	0.776	0.844	0.789	0.833	0.789	0.776	0.782	0.629
	DLRN	0.876 (0.806–0.946)	0.859	0.810	0.896	0.855	0.863	0.855	0.810	0.832	-

*AUC* area under the curve, *CI* confidence interval, *DLR* deep learning radiomics, *DLRN* deep learning radiomics nomogram, *EVC* external validation cohort, *GPTV* gross peritumoral tumor volume, *GTV* gross tumor volume, *IVC* internal validation cohort, *NPV* negative predictive value, *PPV* positive predictive value, *TC* training cohort

* AUCs were compared with the DLRN model using the DeLong test

### Performance evaluation of the DLRN model in patient subgroups

In the IVC and EVC1–3, the AUCs of the DLRN model for predicting apCR before NAC initiation were 0.865–0.909 for patients aged ≤ 45 years and 0.868–0.919 for patients aged > 45 years; 0.876–0.902 for premenopausal patients and 0.873–0.924 for postmenopausal patients; 0.870–0.925 for patients with clinical stage T1–2 and 0.885–0.915 for patients with stage T3–4; and 0.868–0.922 for patients with clinical stage N1 and 0.865–0.903 for patients with stage N2–3 (Fig. S6), indicating that the DLRN model achieved robust performance in all patient subgroups.

### Clinical benefits of the DLRN model

In our study, a total of 811 patients in the IVC and EVC1–3 underwent ALND; following NAC, 375 patients (46.2%) achieved apCR, while 436 patients (53.8%) did not. If ALND was performed on these patients based on the forecasting outcomes of the DLRN model, the rate of unnecessary ALND would decrease from 46.2% to 7.9% (375–64), and the clinical benefit rate would increase from 53.8% to 83.7% (436–679); see Fig. S7.

## Discussion

The accurate prediction of apCR represents a critical clinical need for minimizing surgical overtreatment of the axilla in breast cancer patients undergoing NAC. Here, we employed a quantitative analysis of pre-treatment DCE-MRI images to evaluate the potential of DLR features derived from intra- and peritumoral regions for predicting apCR following NAC in breast cancer. Our results demonstrated that DLR features extracted from GPTV_5_ can serve as stable and accurate predictors. The DLRN model showed robust performance, offering an easy-to-use and personalized apCR prediction tool.

In our study, the apCR rate was 45.7%, exceeding the value of 41.1% reported by Boughey et al [36]. Our cohort’s higher proportion of HER2+ patients, all of whom received anti-HER2 targeted therapy, possibly explains this increase. Our findings align with several investigations showing that the ALN response to NAC correlates with clinical TNM stage and hormone receptor status, including HER2 status [37–40]. Although clinical models incorporating these variables have been constructed, their performance has remained suboptimal, with AUC values spanning 0.686 to 0.746. This underscores the intrinsic limitations of clinicopathological features in predicting apCR and highlights the pressing need for more robust predictive models to reliably assess apCR prior to NAC initiation.

Several studies have reported the feasibility of radiomics and DL methods in predicting apCR following NAC. These approaches aim to provide a more accurate and noninvasive method for preoperative apCR prediction. For instance, Lin et al [19] developed a radiomics model based on multiparametric breast MRI features, achieving an AUC of 0.825. Similarly, Gan et al [20] proposed a radiomics model based on breast tumor MRI features to predict apCR after NAC, with an AUC of 0.751 in an external validation cohort. Zhang et al [41] introduced a radiomics model based on ultrasound images for apCR prediction, reporting AUC values of 0.761 and 0.723 in their validation and external test cohorts, respectively. Gu et al [42] employed pre- and post-NAC ultrasound images to create a DL model, with AUC values of 0.842 and 0.845 in their test and validation cohorts, respectively. However, these models’ clinical applicability is challenged by limitations such as small sample size, limited predictive performance, and low reproducibility. Furthermore, these investigations primarily focused on features extracted from tumors, neglecting potential alterations in the surrounding tissue.

The quantitative combination of imaging features from both the tumor and the surrounding regions can enhance predictive performance [43]. A recent study confirmed the efficacy of integrating intra- and peritumoral radiomics features on ultrasound images to preoperatively predict apCR post-NAC in HER2‐positive breast cancer patients [44]. It is worth noting, however, that this work extracted peritumoral features only within a 3-mm margin, which may fail to capture discriminatory information emanating from a broader peritumoral zone. In the present study, to compare predictive performance across different regions and measure optimal peritumoral region size, we automatically expanded the GTV outward by 3, 5, 7, and 9 mm. Unlike in prior research, we removed the intratumoral mask and extracted features from the incorporated segmentation. Past studies have shown that the GPTV model outperforms models based solely on GTV or gross peritumoral tumor volume, indicating that peritumoral features offer complementary data to intratumoral features [45]. Accordingly, the constructed GPTV model captures biological information from within and around the tumor and streamlines clinical implementation. Notably, we utilized three EVCs to prove the generalizability of the constructed model. Our results showed that the GPTV_DLR model, which included the peritumoral regions, had higher prediction accuracy than the GTV model, verifying that incorporating both intratumoral components and incremental peritumoral information dramatically improves prediction performance. By comparing performance metrics across various GPTV models, we identified the GPTV_5__DLR model as exhibiting the best prediction performance, with an average AUC of 0.876 in the VCs. One possible explanation is that a 5-mm peritumoral expansion strikes the best balance between capturing relevant peritumoral signals and avoiding extraneous background noise.

Furthermore, we developed a DLRN model incorporating the clinical N stage, ER, HER2, and GPTV_5__DLR model to predict apCR post-NAC in breast cancer. This model demonstrated promising performance, with AUCs of 0.885, 0.911, and 0.876 in EVC1–3, dramatically surpassing the clinical model. The DLRN model showed stable prediction performance in subgroup analysis based on age, menopausal status, and clinical N and T stages. We further transformed the DLRN model into an open-source application tool, which, upon inputting corresponding clinical information and DLRN scores, can assist clinicians in calculating the predicted probability of apCR. This tool may aid clinicians in formulating and optimizing treatment strategies, potentially providing more personalized and less invasive therapeutic options for breast cancer patients. By effectively distinguishing high- and low-DLRN-score breast cancer patients, the model can help clinicians determine the need for ALND. This stratification strategy is particularly valuable for sparing false-negative patients from unnecessary ALND, while ensuring that false-positive patients receive the treatment needed to achieve optimal therapeutic benefit.

Our work has some limitations. First, as a retrospective study, it is inevitably subject to choice bias and inherent errors. Second, all ROIs in our study were delineated manually, rendering inter-operator variability unavoidable, which may affect feature reproducibility. To address these issues, future work will focus on implementing and validating a DL-based automated segmentation method and performing quantitative comparisons with manual segmentation to improve the study’s methodological quality. Third, molecular subtype distribution among patients was uneven, with certain subtypes represented by relatively small sample sizes, which may have introduced a degree of statistical bias. To mitigate bias, we intend to further increase specimen size and survey the apCR prediction model performance in patients with varied molecular subtypes. Fourth, due to the relatively short follow-up period for the included patients, we were unable to reliably assess the prognostic value of the DLRN model. Continued prospective follow-up of our study cohort is planned to clarify whether its apCR-based stratification translates into durable clinical benefit.

In summary, the GPTV_5__DLR features derived from preoperative DCE-MRI images serve as reliable noninvasive biomarkers for predicting apCR after NAC in breast cancer patients. Moreover, the DLRN model integrating the GPTV_5__DLR model with clinicopathological predictors further enhances predictive performance. The DLRN model’s prediction results regarding apCR after NAC could assist physicians in optimizing treatment decisions and prevent patients from undergoing unnecessary ALND.

## Supplementary information

ELECTRONIC SUPPLEMENTARY MATERIAL

## Data Availability

The datasets used and/or analyzed during the current study are available from the corresponding author on reasonable request.

## Abbreviations

ALND: Axillary lymph node dissection
apCR: Axillary pathological complete response
AUC: Area under the curve
DCA: Decision curve analysis
DCE: Dynamic contrast-enhanced
DL: Deep learning
DLR: Deep learning radiomics
DLRN: Deep learning radiomics nomogram
GTV: Gross tumor volume
HCR: Handcrafted radiomics
LR: Logistic regression
NAC: Neoadjuvant chemotherapy
pCR: Pathological complete response
ROC: Receiver operating characteristic
ROI: Region of interest
