# Supplementary material for: Multiregional MRI-based deep learning radiomics to predict axillary response after neoadjuvant chemotherapy in breast cancer patients
Source: Insights Imaging. 2026 Jan 26;17:21. doi: 10.1186/s13244-025-02193-1 (PMC12835486; doi:10.1186/s13244-025-02193-1)
Supplement: Supplementary file 1 — ELECTRONIC SUPPLEMENTARY MATERIAL [file 13244_2025_2193_MOESM1_ESM.pdf]

Multiregional MRI-based deep learning radiomics to predict axillary response after neoadjuvant chemotherapy in breast cancer patients

ELECTRONIC SUPPLEMENTARY MATERIAL

Part I. Supplementary Appendix

Part II. Supplementary Tables

Part III. Supplementary Figures

Part I. Supplemental Appendix

Appendix E1. Inclusion and exclusion criteria for patients with breast cancer

Inclusion criteria are as follows: (i) biopsy-proven invasive breast cancer; (ii) completion of standardized NAC followed by ALND; (iii) dynamic contrast enhanced (DCE)-MRI performed within 2 weeks prior to NAC; and (iv) complete clinical and pathological data.

Exclusion criteria included: (i) multifocal or bilateral lesions; (ii) receipt of any treatment prior to NAC; (iii) history of axillary surgery; (iv) suboptimal MRI quality or inconspicuous lesions; and (v) evidence of distant metastasis.

Appendix E2. NAC regimen and clinicopathological data

In this study, patients received six to eight cycles of a taxane based chemotherapy regimen, with some protocols also incorporating anthracycline agents. Patients with human epidermal growth factor receptor 2 (HER2) positive breast cancer were treated with targeted anti-HER2 therapy. The treatment protocol and timeline followed the guidelines provided by NCCN(1) and China Anti-Cancer Association (CACA). The final axillary lymph nodes (ALN) status was determined by surgical pathology. The axillary pathological complete response (apCR) was defined as the complete absence of both micrometastases and macrometastases in the ALNs(2).

Clinicopathological data of enrolled patients were recorded, including age, menstrual status, and clinical T and N stages(3). Histopathological diagnoses were confirmed by two experienced breast pathologists using immunohistochemistry based on the core needle biopsies before NAC. Histological type was classified as invasive ductal carcinoma and others. Estrogen receptor (ER), progesterone receptor (PR), HER2, and Ki-67 index were evaluated. ER and PR were considered positive if more than one percent of tumor cells exhibited staining. Ki-67 expression was categorized as either high or low, using a threshold of 20 percent. HER2 status was determined based on immunohistochemistry scores: scores of zero or one were considered negative, three positive, and equivocal cases with a score of two were further assessed by fluorescence in situ hybridization. Based on molecular receptor expression, breast cancers were classified into three subtypes: hormone receptor positive (HR+) and HER2 negative (HER2-), HER2 positive (HER2+), and triple negative (TN) breast cancer.

Appendix E3. MRI images acquisition

Patients underwent MRI examinations in the prone position. The dynamic contrast enhancement (DCE) protocol comprised one pre and five postcontrast axial image acquisitions using a contrast agent (Gd-DTPA; Bayer Healthcare, Berlin, Germany) injected into the median cubital vein at a dose of 0.1 mmol/kg, followed by 20 mL of normal saline at a rate of 2.0 mL/s.

The Fifth Affiliated Hospital of Wenzhou Medical University (Center 1): Using Dutch Philps Ingenia 3.0T MR scanning system and German Siemens MAGNETOM Area 1.5T MR scanning system, 8-channel dedicated surface coil for breast cancer conventional and enhanced scan. Patients in prone position, bilateral breast naturally overhang in the coil.

The Sixth Affiliated Hospital of Wenzhou Medical University (Center 2): Using American GE Discovery 750w 3.0T with an eight-channel dedicated surface breast coil. Patients in prone position, bilateral breast naturally overhang in the coil.

The Third Affiliated Hospital of Wenzhou Medical University (Center 3): Using

German Siemens MAGNETOM Area 1.5T MR scanning system, 8 channel dedicated surface coil for breast cancer conventional and enhanced scan. The specific scanning scheme is similar to that of the 1.5T scanner in the Fifth Affiliated Hospital of Wenzhou Medical University.

The First Affiliated Hospital of Zhejiang University (Center 4): Using GE Signa HDXT 3.0T MR scanning system, 8 channel dedicated surface coil for breast cancer MRI scan and enhanced scan. Patients in prone position, bilateral breast naturally overhang in the coil. All relevant DCE-MRI sequence parameters are shown in **Table S1**.

Appendix E4. Image preprocessing

To reduce variability caused by differences in MRI acquisition protocols and hospitals, all original MR images were appropriately pre-processed. First, the images were resampled to 1 × 1 × 1 mm^3 (x, y, z) using a linear interpolation algorithm, and the voxel spacing was normalized. Then, a 25 HU bin width was set to discretize the voxel intensity and reduce the noise. Finally, the images were normalized.

Appendix E5. Handcrafted features extraction

The 1218 handcrafted radiomics (HCR) features were obtained from each 3D ROI of each patient using the “Pyradiomics” package in Python (version 3.8.1; https://pyradiomics.readthedocs.io/). The HCR features were extracted, including 18 first-order statistical features, 14 shape features, and 68 texture features, including 22 gray-level co-occurrence matrix (GLCM), 14 gray-level dependence matrix (GLDM), 16 gray-level size zone matrix (GLSZM), and 16 gray-level run length matrix (GLRLM) features. To capture high-throughput image features, voxel intensities were subjected to non-linear transformations (square, square root, logarithm, and exponential). Laplacian of Gaussian (LoG) filtering was applied using sigma values of 1, 2, 3, 4, and 5 mm. Additionally, 8 wavelet decompositions (LLL, LLH, LHL, LHH, HLL, HLH, HHL, HHH) were performed to extract further first-order and texture features. Thus, a total

of 6090 features from five ROIs of different regions per patient were extracted.

Appendix E6. Deep learning feature extraction

In our study, the deep learning (DL) feature extraction process utilized the Vision Transformer (ViT) model, which had been transferred-learned from its pre-training on the ImageNet dataset. The ViT was chosen for its ability to capture global context and its robust performance in image understanding tasks, enabling the effective extraction of complex patterns and structural details that are critical for accurate feature representation(4). To simplify the binary classification, we reduced the fully connected layer of the model from 1000 units to 2 units; this classified the tumors into apCR and non-apCR categories. The 3D images were cropped to separate the tumor ROI, which was then used as the input of the model. Subsequently, the grayscale values of the image were normalized to the range of -1, 1 using min-max scaling. Subsequently, the cropped ROI images were resized to 64×64 pixels using nearest neighbor interpolation to standardize the input of the model. Our learning rate was set as follows:

$$\eta_t^{\mathrm{task-spec}} = \eta_{\mathrm{min}}^{\mathrm{i}} + \frac{1}{2}(\eta_{\mathrm{max}}^{\mathrm{i}} - \eta_{\mathrm{min}}^{\mathrm{i}})(1 + cos(\frac{T_{cur}}{T_i}\pi)) \quad (1)$$

$\eta_{\mathrm{min}}^{\mathrm{i}} = 0, \eta_{\mathrm{max}}^{\mathrm{i}} = 0$, T_i= 500. It stands for the number of iteration epochs, the lowest learning rate, and the maximum learning rate, respectively. In order to guarantee the migration impact, the backbone section employs pretraining settings. OnT_{cur}= $\frac{1}{2}T_i$Fine tune the parameters of the backbone part. Therefore, the learning rate of backbone part is as follows:

$$\eta_t^{\mathrm{backbone}} \begin{cases} 0 & \mathrm{if} T_{cur} \leq \frac{1}{2}T_i \\ \eta_{\mathrm{min}}^{\mathrm{i}} + \frac{1}{2}(\eta_{\mathrm{max}}^{\mathrm{i}} - \eta_{\mathrm{min}}^{\mathrm{i}})(1 + cos(\frac{T_{cur}}{T_i}\pi)) & \mathrm{if} T_{cur} > \frac{1}{2}T_i \end{cases} \quad (2)$$

The models were optimized using the stochastic gradient descent optimizer with an initial learning rate of 0.001 and a sigmoid cross-entropy loss function. The batch size was set to 8, with L2 regularization and early stopping techniques incorporated to prevent overfitting. Model performance was evaluated based on the loss rate. Once the DL model had completed its training, features were extracted from the output of the final fully connected layer to represent the DL features. The code for DL feature

extraction was obtained from the “Onekey AI” platform and is based on PyTorch (version 1.8.0, http://www.medai.icu/). There was a total of 256 DL features extracted from each 3D ROI, totaling 1280 for each patient.

Appendix E7. The z-score normalization and ComBat method

(1) The z-score normalization

To eliminate differences in scale across feature dimensions, all features were standardized using z-score normalization. The standardized formula is as follows:

$$f(x) = \frac{s(x - \mu_x)}{\delta_x}$$

where x is the original intensity, $f(x)$ is the normalized intensity, μ and δ are the mean and variance, respectively, and s is an optional scaling factor (set to 1 by default).

(2) The ComBat batch calibration

ComBaTool was used to normalise the features. ComBaTool, a free online application (https://forlhac.shinyapps.io/Shiny_ComBat/), was used to pool features and minimize inter-scanner variability. With Center 1 (Center 1: 539 patients with 3.0T MRI examination) as the reference batch, other batches (Center 2: 367 patients with 3.0T MRI examination, Center 3: 201 patients with 1.5 T MRI examination, Center 4: 135 patients with 3.0T MRI examination) were combat corrected.

Appendix E8. ICC calculation and feature selection method

(1) Inter- and intra-class correlation coefficients calculation

The reproducibility of each HCR feature was evaluated using inter- and intraclass correlation coefficients (ICCs) to quantify their stability. First, 50 patients were randomly selected from the training cohort of Center 1 and re-segmented by radiologists 1 and 2, who had eight and ten years of expertise in MRI diagnosis of breast diseases, respectively, using the same tools and environment settings. Meanwhile, two adjacent slices (ROI-1 and ROI-2) of the selected breast tumor region slice were re-outlined in 50 patients to assess the robustness of the adjacent slice

features. The time interval between the two manual segmentations by radiologist 1 was 3 months. The ICCs were calculated using a two-way random effects model to determine the inter- and intra-observer reliability. Only HCR features with high reliability (ICC ≥ 0.80) were considered robust and retained for feature selection.

(2) Feature selection method

The screening method for HCR and DL features was divided into the following three steps. First, Spearman correlation analysis was used to identify highly correlated features. This strategy iteratively removed the most redundant feature in the feature set until no feature pairs with a correlation coefficient > 0.9 remained. Second, the Mann–Whitney U-test was conducted per feature to choose features significantly associated with the apCR outcome, with a *P*-value below the 0.05 threshold. Then, dimensionality reduction was conducted utilizing the least absolute shrinkage and selection operator (LASSO) algorithm with 10-fold cross-validation.

Appendix E9. Feature visualization and analysis

For HCR features, we selected the three features with the highest coefficients in LASSO for visualization and generated maps of the selected HCR features to explore their effectiveness in identifying spatial variations in apCR after NAC. Firstly, voxel-level feature mapping, the calculation results of HCR features are mapped back to the corresponding voxel positions of the original 3D images, and the feature volume data is generated. Secondly, pseudo-color overlay, using color mapping (such as Jet and Viridis) to convert feature values into colors, overlays on grayscale images, and adjusts the transparency to retain the anatomical structure. Thus, the pseudo-color image is overlaid on the DCE-MRI image to form a visualization of the HCR features, with blue indicating low feature values and red indicating high feature values.

For DL features, gradient-weighted class activation mapping (GRAD-CAM) was applied to examine significant areas within the DCE-MRI images. Grad-CAM works by quantifying the correlation between category predictions and feature maps from convolutional layers, producing class activation maps for the chosen DL features. This technique was implemented to depict how the segmentation model allocated attention

to different regions in the input images for the selected features.

Appendix E10. METRICS tool

Categories	No.	Items	Weights	Score[f]
Study design	#1	Adherence to radiomics and/or machine learning-specific checklists or guidelines	0.0368	0.0368
	#2	Eligibility criteria that describe a representative study population	0.0735	0.0735
	#3	High-quality reference standard with a clear definition	0.0919	0.0919
Imaging data	#4	Multi-center	0.0438	0.0438
	#5	Clinical translatability of the imaging data source for radiomics analysis	0.0292	0.0292
	#6	Imaging protocol with acquisition parameters	0.0438	0.0438
	#7	The interval between imaging used and reference standard	0.0292	0.0292
Segmentation[a]	#8	Transparent description of segmentation methodology	0.0337	0.0337
	#9	Formal evaluation of fully automated segmentation[b]	0.0225	0
	#10	Test set segmentation masks produced by a single reader or automated tool	0.0112	0.0112
Image processing and feature extraction	#11	Appropriate use of image preprocessing techniques with transparent description	0.0622	0.0622
	#12	Use of standardized feature extraction software[c]	0.0311	0.0311
	#13	Transparent reporting of feature extraction parameters, otherwise providing a default configuration statement	0.0415	0.0415
Feature processing	#14	Removal of non-robust features[d]	0.0200	0.0200
	#15	Removal of redundant features[d]	0.0200	0.0200
	#16	Appropriateness of dimensionality compared to data size[d]	0.0300	0.0300

Categories	No.	Items	Weights	Score[f]
	#17	Robustness assessment of end-to-end deep learning pipelines[e]	0.0200	0.0200
Preparation for modeling	#18	Proper data partitioning process	0.0599	0.0599
	#19	Handling of confounding factors	0.0300	0.0300
Metrics and comparison	#20	Use of appropriate performance evaluation metrics for task	0.0352	0.0352
	#21	Consideration of uncertainty	0.0234	0.0234
	#22	Calibration assessment	0.0176	0.0176
	#23	Use of uni-parametric imaging or proof of its inferiority	0.0117	0.0117
	#24	Comparison with a non-radiomic approach or proof of added clinical value	0.0293	0.0293
	#25	Comparison with simple or classical statistical models	0.0176	0.0176
Testing	#26	Internal testing	0.0375	0.0375
	#27	External testing	0.0749	0.0749
Open science	#28	Data availability	0.0075	0
	#29	Code availability	0.0075	0
	#30	Model availability	0.0075	0
Total METRICS score (should be given as percentage)				97.7%
Quality category[g]				Excellent

[a]Conditional for studies including region/volume of interest labeling

[b]Conditional for studies using fully automated segmentation

[c]Conditional for the hand-crafted radiomics

[d]Conditional for tabular data use

[e]Conditional on the use of end-to-end deep learning

[f]Score is simply the weight if present and 0 otherwise

[g]Proposed total score categories: 0 ≤ score < 20%, “very low”; 20 ≤ score < 40%, “low”; 40 ≤ score < 60%, “moderate”; 60 ≤ score < 80%, “good”; and 80 ≤ score ≤ 100%, “excellent” quality

Appendix E11. The results of deep learning radiomics feature selection

A total of 1642 features were included in each 3D ROI. Through intra- and inter-observer reproducibility analysis of tumor segmentation, HCR features with ICC > 0.80 were identified (GTV, n = 1084; $GPTV_3$, n = 1145; $GPTV_5$, n = 1182; $GPTV_7$, n = 1193; $GPTV_9$, n = 1060), indicating that the handcrafted features had good reliability. Then, representative features were selected from DL features and the above-mentioned robust HCR features using a three-step feature screening method (HCR features: GTV, n = 7; $GPTV_3$, n = 7; $GPTV_5$, n = 12; $GPTV_7$, n = 15; $GPTV_9$, n = 5; DL features: GTV, n = 10; $GPTV_3$, n = 8; $GPTV_5$, n = 14; $GPTV_7$, n = 9; $GPTV_9$, n = 10). Finally, DL radiomics (DLR) features with nonzero coefficients (GTV, n = 11; $GPTV_3$, n = 13; $GPTV_5$, n = 17; $GPTV_7$, n = 13; $GPTV_9$, n = 9) for predicting apCR before NAC were selected to build the DLR models by using a second round of the least absolute shrinkage and selection operator (LASSO) regression analysis. Details of the DLR feature selection procedure are described in **Table S3**.

Appendix E12. Predicted score calculation of the DLR models

The formula for calculating the score of the DL radiomics model is as follows:

Predicted score = $\frac{1}{1+e^{-f(x)}}$

$f(x) = \mathrm{b}_0 + \mathrm{b}_1 \times \chi_1 + \mathrm{b}_2 \times \chi_2 + \ldots + \mathrm{b}_\mathrm{m} \times \chi_\mathrm{m}$

Where b_0 represents the intercept value, b_m represents the coefficients of the features, both of which were calculated by logistic regression, and χ_m represents the feature values. The calculation methods for the DLR models are as follows:

① GTV_DLR model: The formula for calculating the predicted score of the GTV_DLR model is as follows:

$f(x)$ = - 0.401 × DL_32 value

- 0.290 × log_sigma_1_0_mm_3D_glszm_SizeZoneNonUniformityNormalized value

- 0.274 × DL_239 value

+ 0.181 × DL_463 value

- 0.155 × wavelet_LLL_gldm_LargeDependenceHighGrayLevelEmphasis value

- 0.102 × DL_171 value

- 0.236 × wavelet_LHL_glszm_GrayLevelVariance value

+ 0.142 × lbp-3D-k_glcm_ClusterShade value

- 0.058 × DL_395 value

+ 0.257 × original_firstorder_Skewness value

- 0.212 × DL_504 value- 2.318

② GTV_3_DLR model: The formula for calculating the predicted score of the GTV_3_DLR model is as follows:

$f(x)$ = - 0.280 × DL_4 value

- 0.295 × DL_197 value

+ 0.189 × wavelet_LLL_glszm_LargeAreaHighGrayLevelEmphasis value

+ 0.192 × DL_435 value

- 0.612 × wavelet_LHH_glszm_SmallAreaLowGrayLevelEmphasis value

- 0.433 × log_sigma_5_0_mm_3D_glszm_GrayLevelNonUniformity value

- 0.236 × DL_91 value

+ 0.104 × log_sigma_2_0_mm_3D_glcm_ClusterShade value

- 0.136 × log_sigma_5_0_mm_3D_gldm_DependenceVariance value

- 0.216 × DL_501 value

+ 0.011 × original_glcm_Correlation value

+ 0.272 × wavelet_LLL_gldm_LargeDependenceHighGrayLevelEmphasis value

- 0.416 × DL_258 value- 0.261

③ GTV_5_DLR model: The formula for calculating the predicted score of the GTV_5_DLR model is as follows:

$f(x)$ = - 0.295 × wavelet_LLL_glszm_GrayLevelNonUniformity value

- 0.212 × DL_7 value

- 0.095 × wavelet_LLL_gldm_LargeDependenceHighGrayLevelEmphasis value

- 0.119 × wavelet_HLL_glszm_ZoneEntropy value

- 0.018 × DL_124 value

+ 0.199 × DL_217 value

- 0.036 × DL_323 value

+ 0.013 × log_sigma_5_0_mm_3D_glszm_SmallAreaHighGrayLevelEmphasis value

- 0.039 × DL_458 value

- 0.117 × log_sigma_2_0_mm_3D_glcm_ClusterShade value

- 0.136 × log_sigma_3_0_mm_3D_glcm_Corelation value

+ 0.005 × DL_152 value

+ 0.087 × original_firstorder_RootMeanSquared value

+ 0.118 × wavelet_HLH_frstorder_Kurtosis

- 0.138 × DL_135

- 0.289 × DL_23

+ 0.314 × wavelet_LLL_glcm_ClusterShade- 0.208

④ GTV_7_DLR model: The formula for calculating the predicted score of the GTV_7_DLR model is as follows:

$f(x)$ = - 0.375 × DL_55 value

- 0.279 × wavelet_HLL_glszm_GrayLevelNonUniformity value

+ 0.014 × wavelet_LLL_gldm_LargeDependenceHighGrayLevelEmphasis value

- 0.142 × DL_355 value

- 0.163 × DL_460 value

- 0.162 × original_firstorder_Mean value

- 0.018 × log_sigma_5_0_mm_3D_glszm_ZoneEntropy value

- 0.013 × DL_267 value

+ 0.023 × logarithm_gldm_LargeDependenceLowGrayLevelEmphasis value

- 0.140 × log_sigma_4_0_mm_3D_firstorder_10Percentile value

- 0.390× DL_154 value

+ 0.186 × DL_28 value

+ 0.391 × DL_9 value- 3.236

⑤ GTV_9_DLR model: The formula for calculating the predicted score of the GTV_9_DLR model is as follows:

$f(x)$ = - 0.204 × DL_31 value

- 0.200 × wavelet_LLH_glcm_ClusterProminence value

+ 0.090 × DL_199 value

- 0.063 × DL_323 value

- 0.109 × log_sigma_2_0_mm_3D_glszm_GrayLevelNonUniformity value

+ 0.021 × DL_271 value

- 0.019 × DL_136 value

+ 0.013 × DL_241 value

+ 0.052 × original_firstorder_Skewness value- 2.733

References:

1. Gradishar WJ, Moran MS, Abraham J, et al. Breast Cancer, Version 3.2024, NCCN Clinical Practice Guidelines in Oncology. J Natl Compr Canc Netw 2024;22(5):331-357. doi: 10.6004/jnccn.2024.0035

2. von Minckwitz G, Untch M, Blohmer JU, et al. Definition and impact of pathologic complete response on prognosis after neoadjuvant chemotherapy in various intrinsic breast cancer subtypes. J Clin Oncol 2012;30(15):1796-1804. doi: 10.1200/jco.2011.38.8595

3. Giuliano AE, Connolly JL, Edge SB, et al. Breast Cancer-Major changes in the American Joint Committee on Cancer eighth edition cancer staging manual. CA Cancer J Clin 2017;67(4):290-303. doi: 10.3322/caac.21393

4. Jiang X, Zhao H, Saldanha OL, et al. An MRI Deep Learning Model Predicts Outcome in Rectal Cancer. Radiology 2023;307(5):e222223. doi: 10.1148/radiol.222223

Part II. Supplemental Tables

Table S1. DCE-MRI sequence parameters of different MR scanners

Parameters	Philps Ingenia 3.0T	GE Discovery 750w 3.0T	GE Signa HDXT 3.0T	Siemens MAGNETOM Area 1.5T
Repetition time (ms)	4.5	4.0	5.4	4.6
Echo time (ms)	2.1	2.1	2.6	1.8
Flip angle (°)	12	10	12	10
Slice thickness (mm)	1.5	2.0	1.2	1.5
Matrix	512×512	320×320	416×416	320×320
Field of view (cm)	300×300	240×240	340×340	360×360

Abbreviations: DCE-MRI, dynamic contrast enhanced-magnetic resonance imaging.

Table S2. Univariate and multivariate logistic regression analysis of clinicopathological characteristics for apCR to NAC of breast cancer in the TC

Variables	Comparisons	Univariate logistic regression			Multivariate logistic regression*		
		OR	95% CI	*P* value	OR	95% CI	*P* value
Age	> 45 *vs.* ≤ 45	1.358	0.915 - 2.015	0.129			
Menopausal status	Postmenopausal *vs.* Premenopausal	0.924	0.630 - 1.354	0.684			
Histological type	Invasive ductal carcinoma *vs.* Others	0.785	0.410 - 1.506	0.467			
Clinical T stage	T3-4 *vs.* T1-2	1.250	0.843 - 1.856	0.267			
Clinical N stage	N2-3 *vs.* N1	0.298	0.185 - 0.482	< 0.001	0.266	0.157 - 0.450	< 0.001
ER	Positive *vs.* Negative	0.549	0.373 - 0.808	0.002	0.455	0.292-0.709	0.001
PR	Positive *vs.* Negative	0.762	0.520 - 1.116	0.162			
HER2	Positive *vs.* Negative	3.231	2.160 - 4.831	< 0.001	4.700	2.971 - 7.436	< 0.001
Ki-67	Positive *vs.* Negative	1.426	0.947 - 2.146	0.089	1.261	0.792 - 2.007	0.329
Molecular subtypes	HER2+ *vs.* HR+/HER2-	1.892	1.241 - 2.884	0.003	1.554	0.958 - 2.519	0.074
	TN *vs.* HR+/HER2-	1.434	0.813 - 2.530	0.213	1.703	0.888 - 3.268	0.109

Note: *Variables found significant at $P < 0.1$ in univariate analyses were entered into multivariate analyses.

Abbreviations: CI, confidence interval; ER, estrogen receptor; HER2, human epidermal growth factor receptor 2; HR, hormone receptor; OR, odds ratio; PR, progesterone receptor; TC, training cohort; TN, triple negative.

Table S3. Handcrafted radiomics feature and deep learning radiomics feature selection procedure

Regions	Total feature		ICC	Mann–Whitney U test		Correlation analysis		Lasso 1st		Lasso 2nd		Total
	HCR	DL	HCR	HCR	DL	HCR	DL	HCR	DL	HC R	DL	
GTV	1218	256	1084	476	175	211	108	7	10	5	6	11
$GPTV_3$	1218	256	1145	512	151	254	93	7	8	7	6	13
$GPTV_5$	1218	256	1182	537	189	301	127	12	14	9	8	17
$GPTV_7$	1218	256	1193	499	162	282	102	15	9	6	7	13
$GPTV_9$	1218	256	1060	462	133	263	87	5	10	3	6	9

Abbreviations: GPTV, gross peritumoral tumor volume; GTV, gross tumor volume; HCR, handcrafted radiomics; DL, deep learning; ICC, interclass correlation coefficient; LASSO, least absolute shrinkage and selection operator.

Table S4. DeLong test results of $GPTV_5$_DLR model compared with the other DLR models in the TC, IVC, and EVC1-3

DLR models	Cohort	z-value	*P* value
$GPTV_5$ *vs.* GTV	TC	4.124	< 0.001
	IVC	1.843	0.065
	EVC1	2.024	0.044
	EVC2	1.571	0.116
	EVC3	1.157	0.247
$GPTV_5$ *vs.* GTV_3	TC	2.037	0.043
	IVC	0.395	0.693
	EVC1	0.835	0.404
	EVC2	0.950	0.342
	EVC3	0.677	0.499
$GPTV_5$ *vs.* GTV_7	TC	3.527	< 0.001
	IVC	1.350	0.178
	EVC1	2.894	0.004
	EVC2	1.571	0.116
	EVC3	0.881	0.378
$GPTV_5$ *vs.* GTV_9	TC	4.358	< 0.001
	IVC	1.561	0.119
	EVC1	3.339	< 0.001
	EVC2	2.458	0.014
	EVC3	2.342	0.019

Abbreviations: DLR, deep learning radiomics; GPTV, gross peritumoral tumor volume; GTV, gross tumor volume; EVC, external validation cohort; IVC, internal validation cohort; TC, training cohort.

Table S5. The allocated points corresponding to each variable in the DLRN model and the calculation formula of DLRN score

**Variables**	**Assignment**	**Categories**	**Points**
Clinical N stage	0	N1	0
	1	N2-3	12.425
ER	0	Negative	0
	1	Positive	7.008
HER2	0	Negative	15.321
	1	Positive	0
$GPTV_5$_DLR model*	- 100 × $GPTV_5$_DLR model		
Points = Clinical N stage + ER + HER2 + $GPTV_5$_DLR model			
**DLRN score** = -4.667e-06 * points ^3 + 0.000958872 * points ^2 -0.045251122 * points + 0.600489308			

Abbreviations: ER, estrogen receptor; HER2, human epidermal growth factor receptor 2; GPTV, gross peritumoral tumor volume; DLRN, deep learning radiomics nomogram.

Table S6. DeLong test results of clinical model, $GPTV_5$_DLR model, and DLRN model in the TC, IVC, and EVC1-3

Models	Cohort	z-value	*P* value
Clinical *vs.* $GPTV_5$_DLR	TC	-6.726	< 0.001
	IVC	-2.696	0.007
	EVC1	-5.462	< 0.001
	EVC2	-4.335	< 0.001
	EVC3	-3.143	0.002
Clinical *vs.* DLRN	TC	-9.014	< 0.001
	IVC	-3.210	0.001
	EVC1	-6.274	< 0.001
	EVC2	-4.979	< 0.001
	EVC3	-3.234	0.001
$GPTV_5$_DLR *vs.* DLRN	TC	-2.035	0.042
	IVC	-0.389	0.698
	EVC1	-0.612	0.541
	EVC2	-0.555	0.579
	EVC3	-0.484	0.629

Abbreviations: DLR, deep learning radiomics; DLRN, deep learning radiomics nomogram; GPTV, gross peritumoral tumor volume; EVC, external validation cohort; IVC, internal validation cohort; TC, training cohort.

Part III. Supplemental Figures

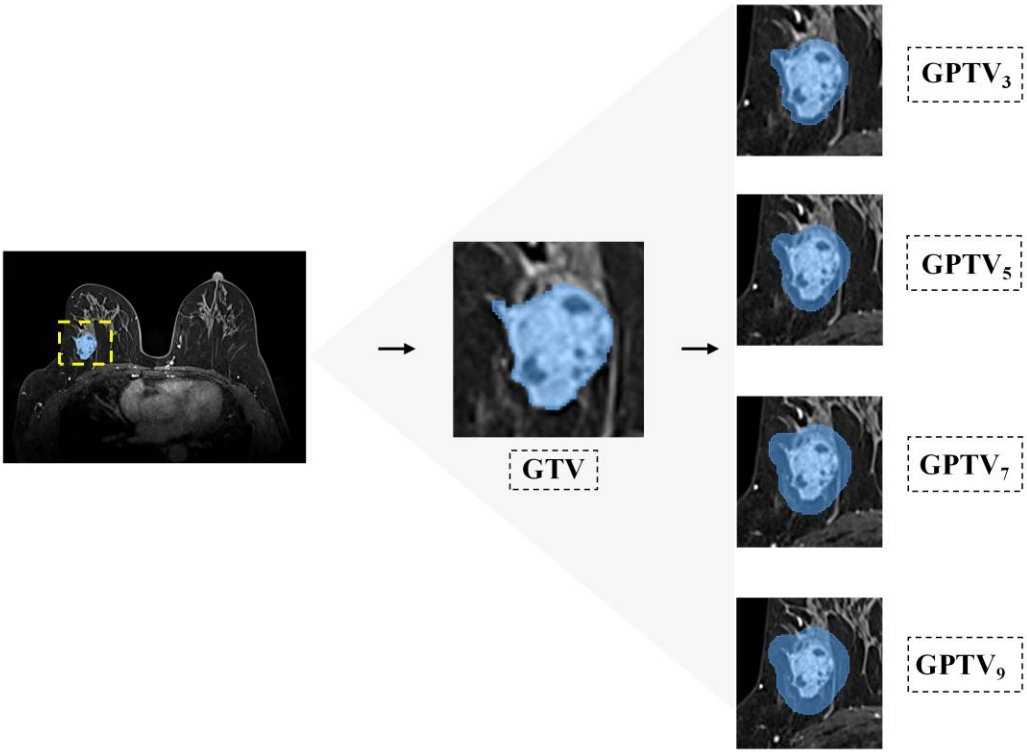

Figure S1. The segmentation process of gross tumor volume (GTV) and gross peritumoral tumor volume (GPTV).

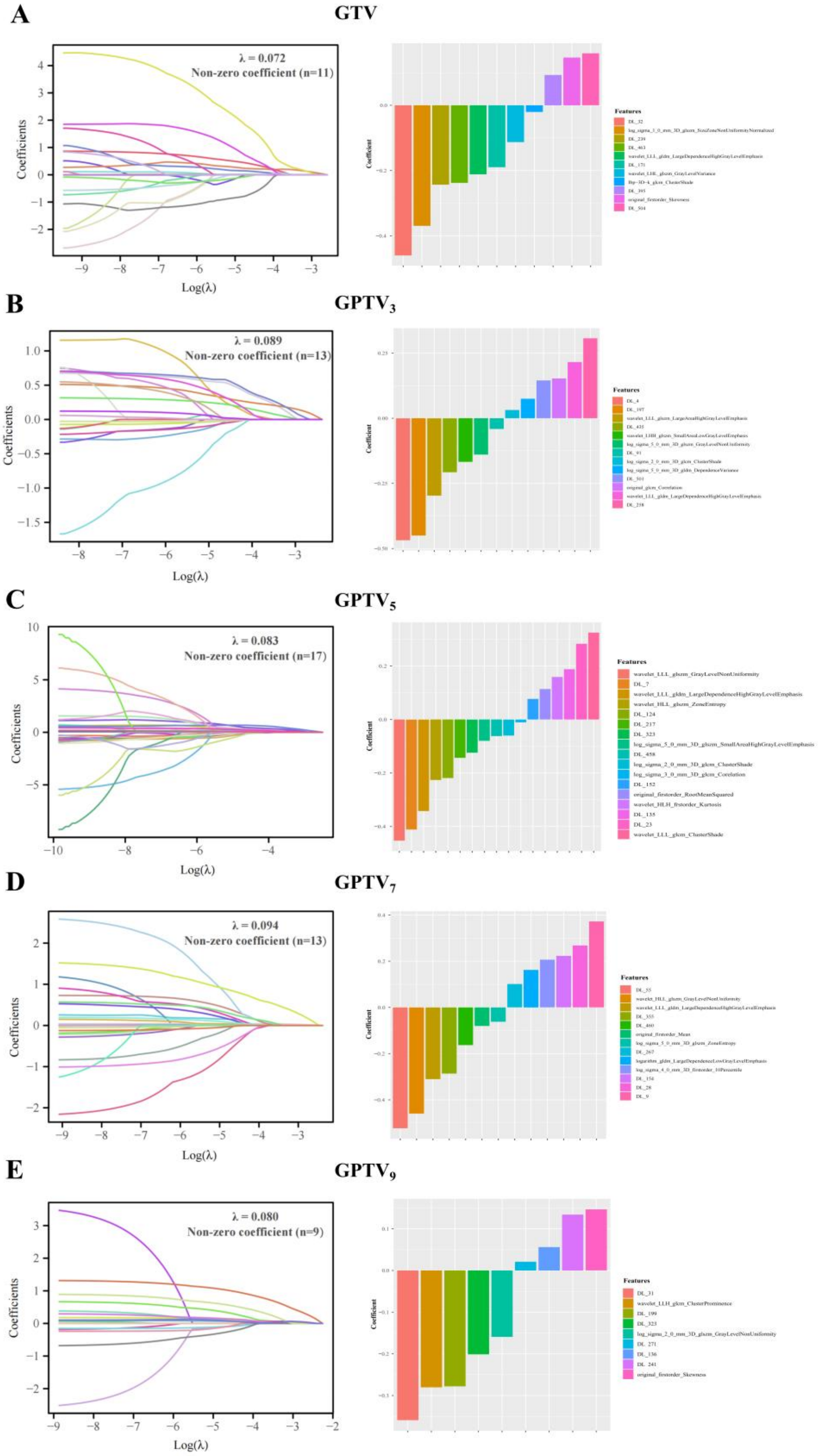

Figure S2. Feature selection (left) and optimal deep learning radiomics feature subsets (right) for gross tumor volume (GTV) (**A**), gross peritumoral tumor volume $(GPTV)_3$ (**B**), $GPTV_5$ (**C**), $GPTV_7$ (**D**), and $GPTV_9$ (**E**) using the least absolute shrinkage and selection operator.

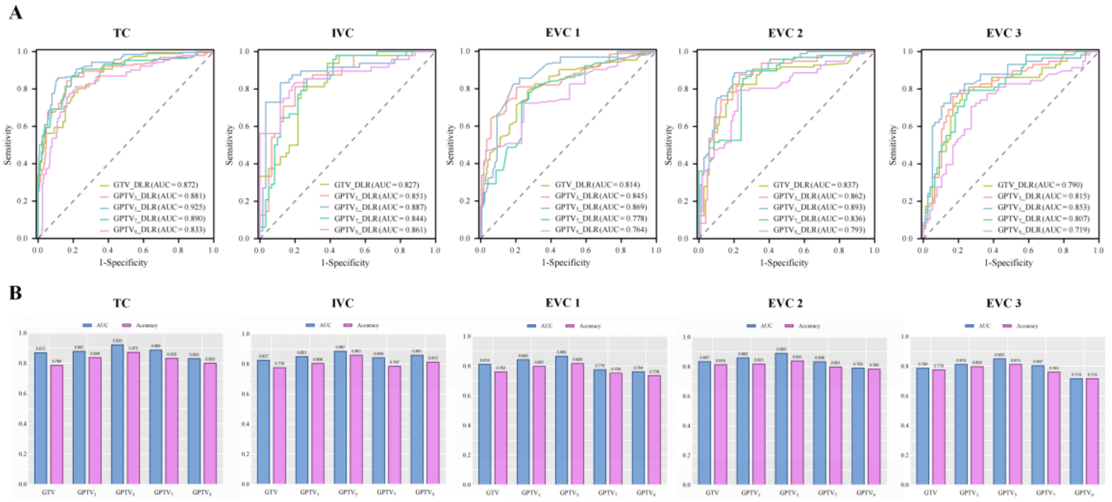

Figure S3. Comparison of the receiver operating characteristic curves (A), area under the curves (AUCs) and accuracies (B) of different deep learning radiomics (DLR) models in the training cohort (TC), internal validation cohort (IVC), external validation cohorts 1-3 (EVC1-3).

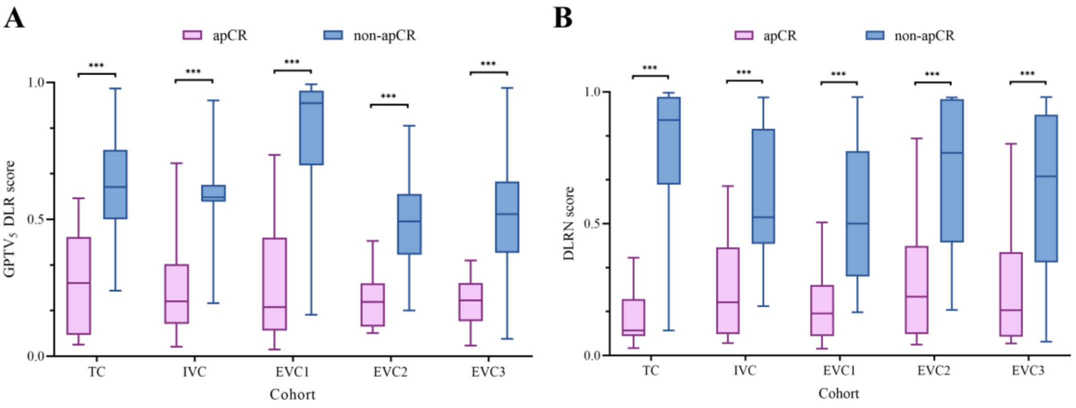

Figure S4. The box plots illustrates the distribution of gross peritumoral tumor volume $(GPTV)_5$ deep learning radiomics (DLR) scores and DLRN scores using the meanmean, maximum, and minimum values. Mann-Whitney U test indicated that there were significant differences in the $GPTV_5$_DLR scores and DLRN scores between non-axillary pathological complete response (apCR) and apCR groups. ***: $P < 0.001$, **: $P < 0.01$, *: $P < 0.05$.

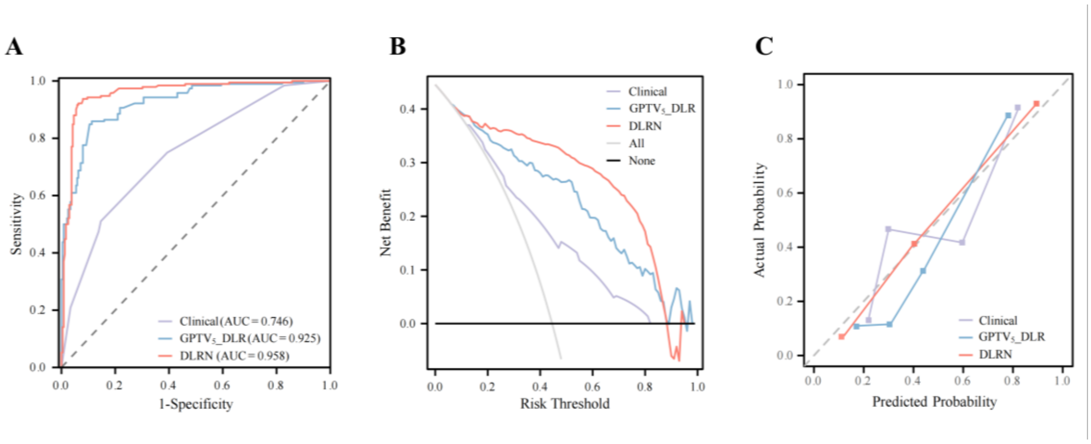

Figure S5. Comparison of the model's performance in the training cohort. (A) Comparison of the receiver operating characteristic curves and area under the curves (AUCs) of the clinical, gross peritumoral tumor volume $(GPTV)_5$_deep learning radiomics (DLR), and DLR nomogram (DLRN) models. (B) Comparison of the decision curves of the clinical, $GPTV_5$_DLR, and DLRN models. (C) Comparison of the calibration curves of the clinical, $GPTV_5$_DLR, and DLRN models.

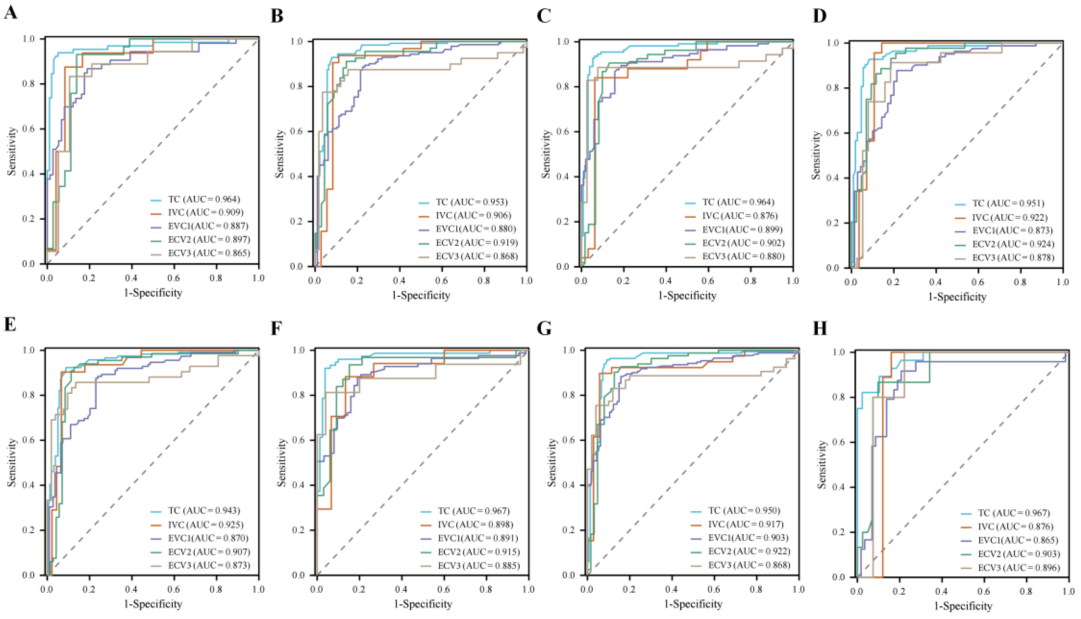

Figure S6. Performance of the deep learning radiomics nomogram (DLRN) model in patient subgroups stratified according to patient age, menopausal status, clinical T and N stages. Receiver operating characteristic curves for (A) age ≤ 45 years, (B) age > 45 years, (C) premenopausal, (D) postmenopausal, (E) clinical T1-2 stage, (F) clinical T3-4 stage, (G) clinical N1 stage, (H) clinical N2-3 stage in the training cohort (TC) (blue line), internal validation cohort (IVC) (orange line), external validation cohort 1 (EVC1) (purple line), external validation cohort 2 (EVC2) (green line), and external validation cohort 3 (EVC3) (grey line) show that the DLRN model combining clinicopathological variables and DLR radiomics model achieved moderate performance in all patient subgroups.

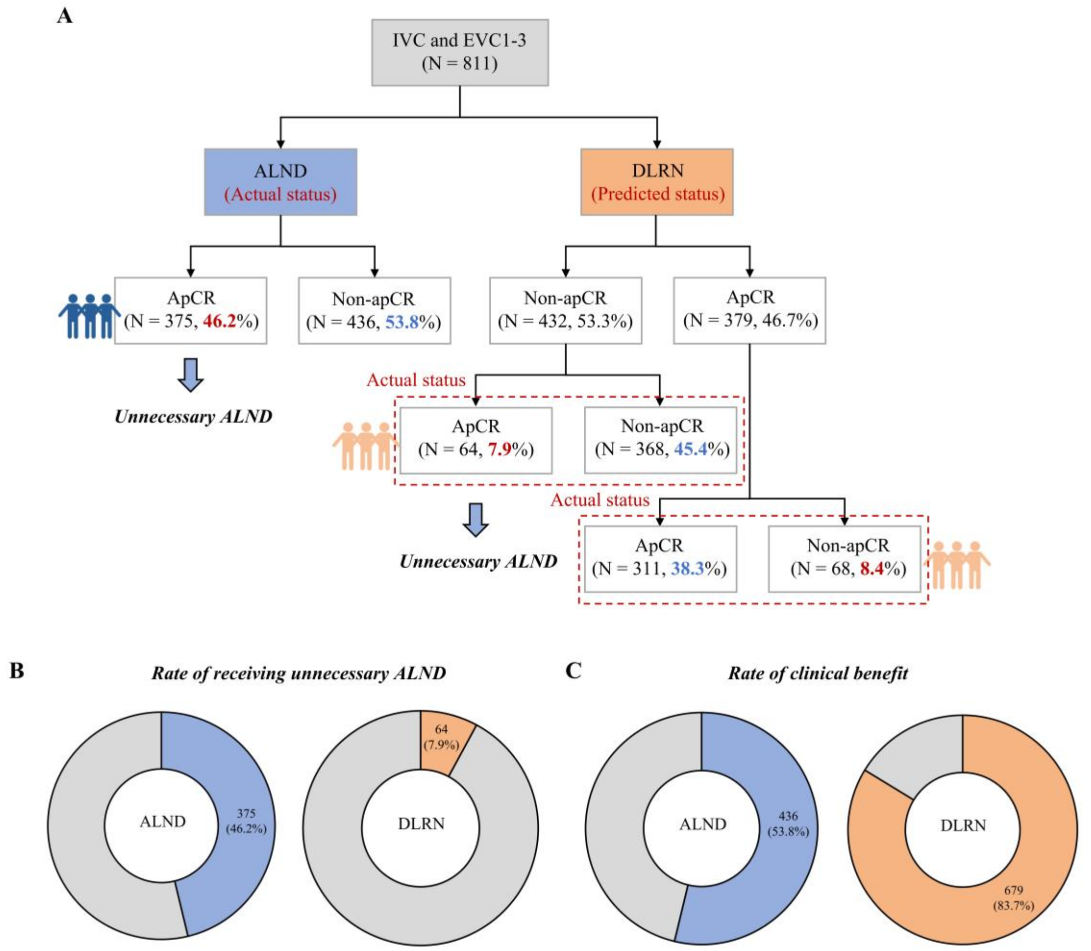

Figure S7. Clinical benefit assessment of deep learning radiomics nomogram (DLRN). (A) Recommendation for axillary lymph node dissection (ALND) according to the DLRN for breast cancer patients with ALN metastasis in the internal validation cohort (IVC) and external validation cohorts (EVCs). (B) The rate of patients receiving unnecessary ALND assessed in two methods. (C) The rate of patients with clinical benefit assessed in two methods.
